# Effectiveness of a multidisciplinary BIOPSYCHOSOCIAL intervention for non-specific SUBACUTE low back pain in a working population: a cluster randomized clinical trial

**DOI:** 10.1186/s12913-019-4810-x

**Published:** 2019-12-12

**Authors:** Romina Raczy Mas, Tomàs López-Jiménez, Enriqueta Pujol-Ribera, María Isabel Fernández-San Martín, Jenny Moix-Queraltó, Elena Montiel-Morillo, Teresa Rodríguez-Blanco, Marc Casajuana-Closas, M. J. González-Moneo, Ester Núñez Juárez, Montse Núñez Juárez, Mercè Roura-Olivan, Raquel Martin-Peñacoba, Magda Pie-Oncins, Montse Balagué-Corbella, Miguel-Ángel Muñoz, Concepción Violan, Anna Berenguera

**Affiliations:** 1 Gerència Territorial de Barcelona, Catalan Institute of Health, c/ Sardenya, 375, Entl, 08025 Barcelona, Spain; 2 Institut Universitari d’Investigació en Atenció Primària (IDIAP Jordi Gol), Gran Via Corts Catalanes, 587, àtic, 08007 Barcelona, Spain; 3 Department of Paediatrics, Gynaecology and Obstetrics and Preventive Medicine, Universitat Autònoma de Barcelona Faculty of Medicine-Bellaterra, 08193 (Cerdanyola del Vallès), Spain; 4 Department of Basic, Evolutionary and Educational Psychology, Universitat Autònoma de Barcelona Faculty of Psychology, Building B. Campus de la UAB, Bellaterra, 08193 Barcelona, Spain; 5 Hospital de la Santa Creu i de Sant Pau, 08041 Barcelona, Spain; 6 Primary Care Centre Sant Martí, SAP Litoral, 08020 Barcelona, Spain; 7 SAP Support to Diagnosis and Treatment, Gerència Territorial de Barcelona, Catalan Institute of Health, 08001 Barcelona, Spain; 8 Unit of Functional Rehabilitation, Department of Rheumatology, Hospital Clínic Universitari, 08036 Barcelona, Spain; 9 Primary Care Centre Sant Andreu, SAP Muntanya, 08030 Barcelona, Spain; 10 Primary Care Centre Sant Antoni, SAP Esquerra, 08015, Barcelona, Spain

**Keywords:** Primary health care, Multidisciplinary biopsychosocial intervention, Non-specific subacute low back pain, Disability, Pain, Quality of life, Cognitive-behavioural therapy

## Abstract

**Background:**

Low back pain (LBP) is a multifactorial condition with individual and societal impact that affects populations globally. Current guidelines for the treatment of LBP recommend pharmacological and non-pharmacological strategies. The aim of this study was to compare usual clinical practice with the effectiveness of a biopsychosocial multidisciplinary intervention in reducing disability, severity of pain and improving quality of life in a working population of patients with subacute (2–12 weeks), non-specific LBP.

**Methods:**

Longitudinal cluster randomized clinical trial conducted in 39 Primary Health Care Centres (PHCC) of Barcelona, with patients aged 18–65 years (*n* = 501; control group = 239; 26 PHCC, intervention group = 262; 13 PHCC). The control group received usual clinical care. The intervention group received usual clinical care plus a biopsychosocial multidisciplinary intervention, which consisted of physiotherapy, cognitive-behavioural therapy and medication. The main outcomes were changes in the Roland Morris Disability Questionnaire (RMDQ), and the minimal clinically important differences. Secondary outcomes were changes in the McGill Pain (MGPQ) and Quality of Life (SF-12) questionnaires. Assessment was conducted at baseline, 3 and 12 months. Analysis was by intention-to-treat and analyst-blinded. Multiple imputations were used.

**Results:**

Of the 501 enrolled patients, 421 (84%) provided data at 3 months, and 387 (77.2%) at 12 months. Mean age was 46.8 years (SD: 11.5) and 64.7% were women. In the adjusted analysis of the RMDQ outcome, only the intervention group showed significant changes at 3 months (− 1.33 points, *p* = 0.005) and at 12 months (− 1.11 points, *p* = 0.027), but minimal clinically important difference were detected in both groups. In the adjusted analysis of the RMDQ outcome, the intervention group improvement more than the control group at 3 months (− 1.33 points, p = 0.005) and at 12 months (− 1.11 points, p = 0.027). The intervention group presented a significant difference. Both groups presented a minimal clinically important difference, but more difference in the intervention group. The intervention group presented significant differences in the MGPQ scales of current pain intensity and VAS scores at 3 months. No statistically significant differences were found in the physical and mental domains of the SF-12.

**Conclusions:**

A multidisciplinary biopsychosocial intervention in a working population with non-specific subacute LBP has a small positive impact on disability, and on the level of pain, mainly at short-term, but no difference on quality of life.

**Trial registration:**

ISRCTN21392091 (17 oct 2018) (Prospectively registred).

## Background

Low back pain (LBP) is a common health problem that affects approximately 80–85% of the general population at least once in their lifetime and has a global prevalence between 17 and 32%, of which 11–12% are disabled by this condition [1, 2]. In a recent survey conducted in Spain, LBP was highly prevalent (50.9%) at all ages, but especially in the working age population (18–65 years old) [3].

The Global Burden of Disease 2010 ranks LBP amongst the top ten causes of DALYs (disability-adjusted life years) [2]. Consequently, LPB is associated with a huge individual and societal burden and remains a frequent reason for medical consultation globally [4]. In Spain alone, LBP generates over 2 million annual consultations in primary care (ENSE 2011/12) [5].

Despite the wide range of treatments and health-care resources devoted to LBP, back-related disability and burden have increased, in recent years [6]. A study carried out in 36 Primary Health Care Centres (PHCC) in Spain showed that despite guideline-based management, the pain continued in 37% and had worsened in 10% of patients after two months [7]. The natural history of LBP can be extremely variable and recurrences are common, with, approximately 65% of patients still experiencing pain one year after the onset of this condition [8, 9].

It is generally accepted that subacute LBP occurs after a period of at least 6 months without LBP, and that it has a duration between 2 and 12 weeks [10]. Research conducted in Spain reported changes in disability, pain and quality of life after 2 weeks of LBP [11]. Once the subacute episode has been established, early interventions are recommended to avoid deterioration, even if it is considered that approximately one third of patients have a favourable evolutions [6].

Compared to no treatment and to other guideline recommendations, recent evidence-based studies support a multidisciplinary approach to ameliorate LBP [6]. Accordingly, it has been suggested that the timely integration of multidisciplinary treatment strategies that include physiotherapy, cognitive-behavioural therapy and medication for patients with non-specific subacute LBP, might reduce the individual and social impact [12]. Following a systematic review by Kamper et al. (2014), who adopted the term ‘multidisciplinary biopsychosocial rehabilitation’ to integrate education and physiotherapy with cognitive-behavioural psychology with the aim to improve disability and function, [9] the current NICE guidelines (2016) recommend early multidisciplinary management [13].

Lastly, the main objective of the current study was to evaluate the change in disability using the validated Spanish version of the Roland Morris Disability questionnaire (RMDQ) across the intervention and its association with minimal clinically important differences. The second goal were to assess changes in pain intensity using the McGill Pain Questionnaire (MGPQ) and in quality of life as measured by the Short Form 12-Item (SF-12).

## Methods

### Design

An analyst-blinded longitudinal cluster randomized controlled clinical trial was conducted. Patients with non-specific subacute LBP treated with a multidisciplinary approach (intervention group) were compared with a control group receiving only usual clinical care. (Current Controlled Trials identifier: ISRCTN21392091) (17 oct 2018) (Prospectively registred). The study protocol has been previously published [12].

### Setting

The trial was conducted in the primary care setting. A total of 39 PHCCs located in Barcelona and its greater metropolitan area participated in the project.

### Study population

Patients were included if they presented LBP lasting between 2 and 12 weeks, and if they did not have a history of LBP during the 6 months prior to the current episode [10, 14]. Participants were active workers, aged between 18 and 65 years, they had to understand Catalan or Spanish and were required to be contactable for at least twelve months after the onset of the study. Exclusion criteria were as follows: patients unwilling to participate; LBP that coexisted with cognitive impairment or psychiatric disorders; other causes of disability which impeded responding to the questionnaires; pregnancy and breast-feeding; physical problems in the preceding 3 months; and a diagnosis of fibromyalgia. In addition, the GP had to certify that no signs or symptoms frequently associated with specific LBP or potentially severe illnesses were present. Detailed information about the recruitment procedure has been published elsewhere [12].

### Randomization (see flowchart-Fig. 1)

Randomization was by cluster, and the randomization unit was the PHCC. A cluster design was used because the intervention was delivered to groups and to minimize contamination. After the PHCC agreed to participate, they were allocated either to the control or the intervention group. During recruitment, the GPs of the PHCC, who knew about the allocation (intervention or control), identified the patients consulting for new episodes of subacute LBP. The patients who met the inclusion criteria were invited to participate, without knowing the allocation of their PHCC. All patients signed the informed consent form.

**Fig. 1:**
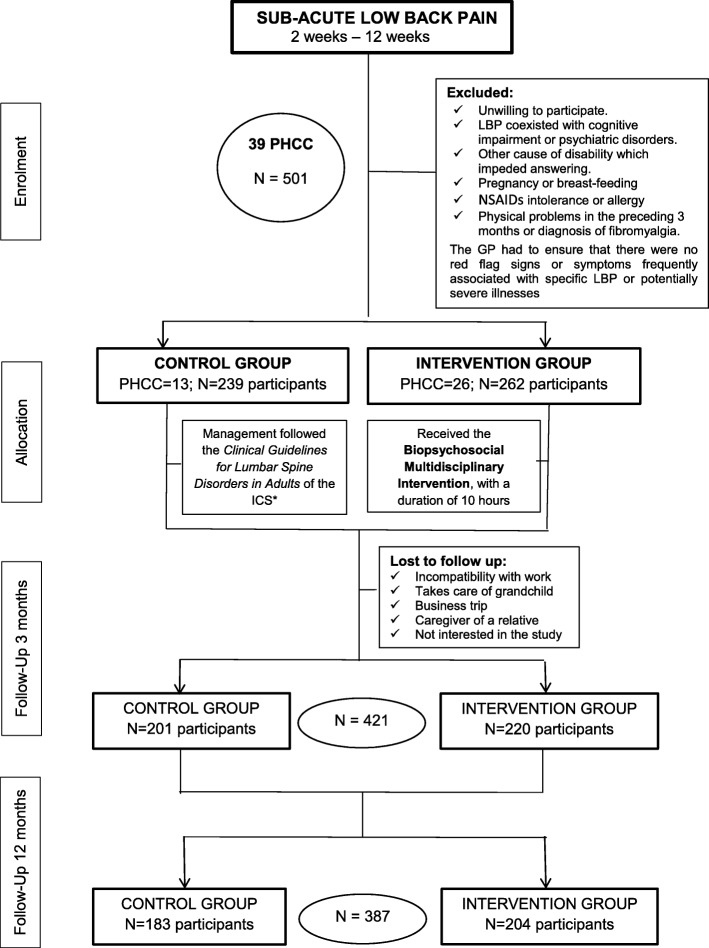
Study Flow chart. Notes: PHCC = Primary Health Care Centres; GP = General practitioner; ICS = Catalan Institute of Health. LBP = Low Back Pain

### Intervention design

Table 1 shows the treatment components of the intervention and the control groups. Both groups received guideline-based pharmacological treatment. Participants in the control group received usual clinical care, based on the *Clinical Guidelines for Lumbar Spine Disorders in Adults* published by the Catalan Institute of Health [15]. Patients allocated to the intervention group received the same care described for the control group, plus the Spanish version of the educational booklet “*The Back Manual”* [16, 17] and some audio visual materials.

**Table 1:** Components of the biopsychosocial multidisciplinary intervention and usual care

		OBJECTIVE	THEORY PROGRAM	PRACTICAL PROGRAM
INTERVENTION GROUP	GP + Nurse 2 h	Answer queries, demystify concepts about LBP and promote adherence to the intervention	Basics of anatomy and biomechanics of the spine	Pain mechanisms, types, causes and susceptibility factors. Healthy life habits, concerns and beliefs about LBP.
	Physiotherapist 4 h	Provide tools on exercises/postures to avoid pain and improve quality of life	Body posture, ergonomics and benefits	Relaxation exercises (breathing), body awareness and postural control
	Psychologist 4 h	Provide participants with cognitive-behavioural therapy techniques.	Influence of cognition, emotions and behaviour in pain	Relaxation guidelines, cognitive restructuring and time management. Assertiveness and problem solving, life values.
CONTROL GROUP	**Clinical Practice Guidelines for Lumbar Spine Disease in Adults** • Patient education, give reassuring and positive information about the benign nature of LBP, offer written information including specific advice. • Advise avoiding bed rest and encourage the person to be physically active and continue with normal activities as far as possible. • Consider offering a structured physical exercises program tailored to personal preferences. • Physical exercise should be introduced gently at first (walking, cycling and swimming) and progressively increased in intensity. • Recommend attendance to the “Back school” after six weeks to those patients who have resumed their daily tasks. • Prescribe pharmacological treatment according to established guidelines.			

*GP* General practitioner, *LBP* Low Back Pain

The intervention was conducted by a GP and/or nurse, a psychologist and a physiotherapist. The programme lasted a total of 10 h, as explained in Table 1. Sessions took place during the week and lasted between 90 and 120 min. To maximise participant adherence to the group sessions, different times were offered. Each group included between 6 and 12 participants and some PHCCs had more than one group receiving the same intervention. To guarantee the standardisation of the group sessions, only one qualified psychologist and one physiotherapist with expertise in group interventions implemented the intervention in all PHCCs. At the end of the study, the control group also received the educational booklet and the audio-visual material.

### Outcomes measures

The main outcome measurement was change in disability as measured by the *Roland Morris Disability Questionnaire* (RMDQ) [18], translated and validated into Spanish [19] (scale 0–24; lower scores indicate less disability). A minimal clinically important difference in disability under 2.5 RMDQ points compared to the baseline value for subacute and chronic patients was considered negligible [20, 21].

Secondary outcome measurements were: intensity of pain, as evaluated with the Spanish version of the *McGill Pain Questionnaire* (MGPQ) (*McGill Pain Questionnaire*, Melzac, 1975) [22, 23], which assesses 3 parameters with 3 dimensions (sensorial, affective and evaluative): Total Intensity Score (scale 0–14), Current Intensity Score (scale 0–5) and Visual Analogical Scale (VAS, scale 0–10); and the mental and physical health-related quality of life, measured with the Spanish version of Short Form 12 version 1 [24] (SF-12, scale 0–100; lower scores indicate worse health related quality of life).

The main independent variable was the intervention arm: biopsychosocial multidisciplinary intervention, or usual clinical care.

### Data collection and follow-up

All participants were invited to attend the PHCC for outcome assessments. They were assessed at baseline and at 3 and 12 months. To maximise patient’s adherence and to avoid loss of participants, patients received a phone call at 6 months. Detailed socio-demographic and clinical variables have been published elsewhere [12].

For each assessment, the same two expert psychologists made up to three phone calls at different times during the day to book the appointments and performed the outcome measures by interviewing the participant, collected information by reviewing medical records, contacted the patient’s GP to inquire about their development (compliance and factors associated with low back pain) and answered questions about the study. A senior psychologist specialised in pain management conducted the intervention.

### Sample size

The sample-size was calculated based on change in RMDQ at three months of follow up. To allow for the cluster randomization by PHCC, we considered an intraclass correlation coefficient of 0.05. In order to detect a difference of 2.5 points between the two intervention arms with a standard deviation of 5.7, an alpha error of 0.05, a beta error of 0.20, and a 20% dropout rate, a sample size of 348 subjects was required per intervention arm, with a the total number of PHCCs of 36. PASS 15 “*Test fot Two Means in a Cluster-Ramndomized Design” module* (Utah, USA, ncss.com/software/pass) was used to calculate sample size.

### Statistical analysis

Data were analysed in accordance with CONSORT guidelines, extension to cluster randomized trials, and based on an intention-to-treat principle. The analysis was performed at the individual level using cluster data methods [25].

The intervention effect at each follow-up was assessed using the change (follow-up minus baseline) in the intervention group minus the change in the control group in the outcomes.

To address potential biases due to incomplete follow-up, multiple imputation by chained equations with 100 imputed datasets was applied to outcomes and covariates [26–28]. Estimates from each imputed dataset were combined following the rules outlined by Rubin [29]. After imputation, the distribution of observed and imputed values was practically equal.

Multivariate regression analysis of each outcome variable was performed for the imputed datasets, taking into account the cluster effect in the models. We conducted linear or logistic mixed-effects model and linear or logistic regression adjusting the standard error for the cluster effect of the PHCC. The final models were adjusted for age, gender, baseline outcome measurement, and the significant confounder and significant interaction variables. We used mixed models and the function “mi estimate” in Stata. In these models, we added the variable PHCC as a cluster/multilevel effect. The linear mixed model was used in the cluster data, with two models for each time-point comparing changes at 3 months and at 12 months with baseline data.

Statistical significance was set at *P* < 0.05 (2-tailed). The analyses were performed using Stata/SE version 14.2 for Windows (Stata Corp. LP, College Station, TX, USA).

## Results

A total of 501 subjects were included in the study; 262 subjects (13 PHCC) were allocated to the intervention group and 239 subjects (26 PHCC) to the control group. After 3 and 12 months, 421 (84%) and 387 (77.2%) participants provided data, respectively. The losses were due to work incompatibility, caregiving duties and lack of interest in the study (see Flowchart, Fig. 1). In general patients who dropped out were significantly younger.

Mean age of participants at baseline was 46.8 (SD: 11.5) years and 64.7% were women. Table 2 shows baseline socio-demographic characteristics and clinical variables, with no statistically significant differences between groups.

**Table 2:** Baseline socio-demographic characteristics and clinical variables

	Total	Control group	Intervention group
No. of PHCC / No. of patients	39/501	26/239	13/262
Socio-demographic characteristics:			
Age (years), *mean (SD)*	46.8 (11.5)	46.4 (11.1)	47.2 (11.9)
Sex (female), *n (%)*	324 (64.7)	145 (60.7)	179 (68.3)
Educational level, *n (%)* (*n* = 499)			
▪ Illiterate or primary school only	122 (24.4)	61 (25.6)	61 (23.4)
▪ Secondary school	274 (54.9)	134 (56.3)	140 (53.6)
▪ University	103 (20.6)	43 (18.1)	60 (23.0)
Paid job (yes), *n (%)*	369 (73.7)	188 (78.7)	181 (69.1)
Clinical variables:			
Body mass index (kg/m^2^) classification (*n* = 500), *mean (SD)*			
▪ Normal weight	222 (44.4)	105 (43.9)	117 (44.8)
▪ Overweight	195 (39.0)	94 (39.3)	101 (38.7)
▪ Obesity	83 (16.6)	40 (16.7)	43 (16.5)

Abbreviations: *SD* standard deviation, *LBP* Low Back Pain, *PHCC* Primary Health Care Centre. Data are mean (SD) or n(%)

Table 3. In the adjusted analysis of the RMDQ outcome, the intervention group improvement more than the control group at 3 months (− 1.33 points, 95% CI: − 2.22 to − 0.45, *p* = 0.005) and at 12 months (− 1.11 points, 95% CI: − 2.08 to − 0.13, *p* = 0.027). The intervention group presented a significant difference. A minimal clinically important difference was achieved in both groups, with a difference over 3.5 points in the intervention group compared with baseline at each time-point (3.8 RMDQ points at 3 months and 5.1 RMDQ points at 12 months).

**Table 3:** Changes in the Roland-Morris Disability, McGill Pain and SF-12 questionnaires between groups at follow-up (*N* = 501)

	Control Group (*n* = 239)		Intervention Group (*n* = 262)		Difference (95%CI) between group (IG - CG)			
	Value	Difference*(95% CI)	Value	Difference*(95% CI)	change IG - CG	*P*-value	Adjusted difference**	P-value
RMDQ, *mean (SD)*								
Baseline	9.9 (5.3)		10.0 (5.2)					
three months	7.4 (5.5)	−2.3 (−3.1 to −1.6)	6.2 (4.9)	−3.8 (−4.5 to −3.2)	−1.5 (− 2.5 to −0.5)	0.003*	−1.33 (− 2.22 to − 0.45)	0.005*
12 months	6.0 (5.7)	−3.8 (− 4.8 to − 2.9)	5.1 (4.9)	−5.1 (−5.8 to − 4.3)	− 1.2 (− 2.4 to − 0.0)	0.043*	−1.11 (− 2.08 to − 0.13)	0.027*
MPQ								
Total intensity score, *mean (SD)*								
Baseline	6.5 (3.1)		6.7 (3.1)					
three months	4.6 (3.6)	−1.8 (−2.3 to − 1.3)	4.0 (3.6)	− 2.7 (− 3.2 to − 2.2)	− 0.9 (− 1.6 to − 0.1)	0.022*	−0.49 (− 1.39 to 0.42)	0.294
12 months	3.6 (3.6)	−2.8 (− 3.3 to − 2.2)	3.1 (3.2)	−3.6 (− 4.1 to − 3.0)	− 0.8 (− 1.6 to 0.0)	0.040*	−0.69 (− 1.41 to 0.02)	0.058**
Current Intensity score *mean (SD)*								
Baseline	2.6 (1.1)		2.5 (1.2)					
three months	1.7 (1.5)	−0.9 (− 1.1 to − 0.7)	1.3 (1.4)	−1.2 (− 1.4 to − 1.0)	−0.3 (− 0.6 to 0.0)	0.083	−0.32 (− 0.63 to − 0.02)	0.040*
12 months	1.6 (1.4)	−1.1 (− 1.3 to 0.8)	1.4 (1.3)	−1.1 (− 1.3 to − 0.9)	0.0 (−0.3 to 0.3)	0.854	−0.18 (− 0.43 to 0.08)	0.162
VAS, *mean (SD)*								
Baseline	5.9 (2.3)		5.8 (2.3)					
three months	4.1 (3.3)	−1.8 (−2.2 to − 1.3)	3.2 (3.2)	− 2.7 (−3.1 to − 2.2)	−0.9 (− 1.6–0.3)	0.004*	−0.77 (− 1.53 to − 0.01)	0.046*
12 months	3.9 (3.2)	−2.0 (− 2.5 to − 1.5)	3.6 (3.0)	−2.3 (− 2.7 to − 1.9)	− 0.3 (− 0.9 to 0.4)	0.404	−0.27 (− 0.88 to 0.34)	0.374
SF-12 Physical health, *mean (SD)*								
Baseline	40.7 (9.3)		41.9 (9.0)					
three months	45.3 (9.8)	4.2 (2.7 to 5.6)	46.5 (8.7)	4.5 (3.2 to 5.8)	0.4 (− 1.6 to 2.3)	0.716	0.55 (− 1.19 to 2.29)	0.520
12 months	46.2 (9.5)	5.0 (3.3 to 6.7)	47.0 (8.9)	4.9 (3.5 to 6.3)	−0.1 (−2.3 to 2.1)	0.922	0.53 (−1.20 to 2.27)	0.532
SF-12 Mental health**,** *mean (SD)*								
Baseline	42.3 (12.4)		43.4 (12.8)					
three months	45.0 (13.2)	2.6 (0.7 to 4.6)	48.8 (12.0)	5.1 (3.4 to 6.9)	2.5 (−0.1 to 5.0)	0.061	2.56 (− 0.33 to 5.45)	0.082
12 months	47.0 (11.9)	5.0 (2.9 to 7.1)	48.9 (11.2)	5.5 (3.6 to 7.5)	0.5 (−2.3 to 3.4)	0.707	1.48 (−0.86 to 3.83)	0.206

Abbreviations: *SD* standard deviation, *CI* confidence interval, *RMDQ* **Roland-Morris Disability Questionnaire** (scale 0–24; lower scores indicate less disability), *MGPQ* **McGill pain questionnaire**; 3 dimensions (sensorial, affective and evaluative) with Total Intensity Score (scale 0–14), Current Intensity Score (scale 0–5) and Visual Analogical Scale (VAS, scale 0–10); **SF-12** = 12-item short-form health survey version 1 (scale 0–100; lower scores indicate worse health related quality of life). * Differences were calculated between follow-up and baseline measurements. Mean differences are shown for quantitative outcomes and percentage differences for dichotomous outcomes. **All models were adjusted for the score at baseline, significant confounders and significant interaction variables. Intervention group minus usual care group, mean differences are shown for quantitative outcomes and odds ratios for dichotomous outcomes. Intervention group minus usual care group, mean differences are shown for quantitative outcomes and odds ratios for dichotomous outcomes. Total Intensity Score, VAS Pain Score and Mental Health were estimated with a mixed model considering the PHCC as random effect

Regarding the level of pain in the adjusted analysis, a marginal difference was observed at 12 months in total intensity, in the intervention group (− 0.69 points; 95% CI: − 1.41 to 0.02; *p* = 0.058). However, the intervention group presented a significant differences at 3 months for current intensity score (− 0.32 points; 95% CI: − 0.63 to − 0.02; *p* = 0.040) and for VAS score (− 0.77 points; 95% CI: − 1.53 to − 0.01; *p* = 0.046).

The outcome of SF-12 increased in both groups during the follow-up period, but no statistically significant differences between groups on the physical and mental health domains were observed.

## Discussion

The aim of this study was to evaluate the effectiveness of a multidisciplinary biopsychosocial intervention in an active population with non-specific subacute LBP. The results shown in Table 3 were obtained with multiple imputation, although similar values were obtained ​​without multiple imputation (data not shown). The main results indicate statistically significant differences regarding disability and pain intensity, with a small effect in the intervention group. Although greater in the intervention group, minimal clinically important differences in disability were achieved in both groups. No differences were observed regarding quality of life.

The results of this trial agree with some studies on subacute and chronic LBP, where moderate quality evidence showed efficacy in contrast with a non-multidisciplinary rehabilitation [8, 10]. Our findings provide new information on the role of multidisciplinary biopsychosocial interventions delivered in groups in the primary care setting.

The initial study sample consisted of 696 participants and 36 PHCCs. Eventually, 501 participants from 39 PHCCs were recruited, since the period of recruitment was not extendable. Recruitment bias was detected, apparently the GPs of the intervention PHCC were more motivated to recruit. To balance the number of participants, more PHCCs were included in the control group.

The minimal clinically important difference for disability was over 2.5 RMDQ points. Kovacs et al. (2007) showed that an improvement in disability below 2.5 RMDQ points compared to their baseline in each group was clinically irrelevant in patients with subacute and chronic LBP. When analyzing each time-point, differences in disability were greater in the short term in both groups. A meta-analysis of LBP showed noticeable short-term improvement during the first six weeks with multiple treatments, but beyond this time improvement slowed [30]. Some other studies reported that compared with usual care, multidisciplinary rehabilitation reduces pain intensity and disability, mainly short-term (< 3 months) [31]. Specifically, Fritz (2015) evaluated the outcomes of early physiotherapy versus usual care, with moderate short-term improvement in disability and pain reduction, but no statistically significant improvement after 1 year [32].

We believe that the perceived improvement of disability may be sufficient for some participants but not all. Considering that over 50% of participants in this intervention were women, some studies indicated that patients with greater disability and worse quality of life were frequently women who suffered also from somatic and mood co-morbidities and perceived higher levels of pain [33–35]. In addition, Chow and colleagues pointed at factors that had been associated with persistent disabling LBP, namely maladaptive pain coping behaviour, high baseline functional impairment, and low general health status [36].

According to the MGPQ, short-term pain reduction was observed to be slightly better in the intervention group. However, some research indicates that minor improvements might be underestimated by patients with LBP that return to their activity or work when non-disabling pain persists [37, 38]. Other studies confirm positive results in pain reduction within six weeks, and emphasize the benefits of a multidisciplinary intervention [39]. For instance, Kamper and colleagues (2015) found that multidisciplinary biopsychosocial rehabilitation for subacute LBP was more effective than other physical interventions. In contrast, a systematic review conducted by the Cochrane Database 2017 did not find any evidence that this type of intervention was more effective than other treatments for subacute LBP [4].

The positive effect on disability and pain intensity was not corroborated by the results regarding quality of life. The lack of improvement in quality of life might be explained different levels of disability or pain at baseline. For some patients, the effect at follow-up might not achieve their expectations of improvement. In patients with levels of disability or pain that are moderate or low at baseline, the effect of the intervention will have a better perceived impact. Other findings suggest that early active physiotherapy can lead to improved outcomes in global health perception [34]. Interestingly, some authors define the relationship between physical activity and the risk of chronic LBP as a U-shaped distribution, i.e., both too little and excessive activity presented increased risks of chronic LBP and worse quality of life [36, 40, 41]. Other factors that intervene in the evolution of LBP include explaining the natural history of this condition and the implementation of the guidelines that seek to relieve or minimize pain.

### Further research

Further research on complex interventions in LBP should consider the UK Medical Research Council (MRC) framework, which consists of several phases that can be iterated and that use qualitative and quantitative methods. This methodology includes the perspective of the patient throughout the study in order to design the intervention based on the population needs, preferences and experiences. The MRC methodology aims to design feasible, effective and sustainable interventions for the primary health care setting.

Finally, we must encourage the collaboration of primary care professionals and the community in everyday clinical practice and in large scale, multidisciplinary interventions.

### Limitations of the study

The number of missing data was similar for both groups at three and 12 months of follow-up. However, the following were more likely to drop out of the study: younger people; people with a lower consumption of analgesics; and at 12 months follow-up, patients with lower family burden and better jobs.

Some confounding factors might limit the conclusions of this study. For instance, the differences in the profile of patients, since they were allocated by PHCC and socioeconomic status was not considered.

One of the most important limitations of the study was recruitment, since more PCHH were allocated to the control group than the intervention group.

## Conclusions

The main conclusion of this study is that a multidisciplinary biopsychosocial intervention in a working population with non-specific subacute LBP has a small positive effect on disability and intensity of pain. Although greater in the intervention group, minimal clinically important differences were achieved in both groups. The results did not show any differences on quality of life.

The results of this biopsychosocial multidisciplinary intervention agree with previous studies, which also show limited effectiveness. The main advantage of delivering an intervention from a PHCC is geographical proximity, which should result in higher adherence. In patients with pain, travelling long distances has usually a negative physical and financial impact. Finally, the constellation of symptoms presented by patients with LBP still constitutes a challenge for medical and surgical decision making.

## Data Availability

The datasets used during the current study are available from the corresponding author on reasonable request.

## Abbreviations

GP: General practitioner
LPB: Low back pain
MGPQ: McGill pain questionnaire
NSAIDs: Non-steroidal anti-inflammatory drugs
PHCC: Primary health care centre
QoL: Quality of life SF12
RMDQ: Roland morris disability questionnaire
VAS: Visual analogical scale
